# Enantioselective Properties of Neglected Drug Nifurtimox on Polysaccharide and Macrocyclic Glycopeptide Chiral Stationary Phases by Green HPLC Separation Methods

**DOI:** 10.1002/jssc.70258

**Published:** 2025-09-08

**Authors:** Amanda Mohr, Érika Segala, Gustavo Machado das Neves, Vera Lucia Eifler‐Lima, Martin Steppe

**Affiliations:** ^1^ Programa de Pós‐Graduação em Ciências Farmacêuticas Universidade Federal do Rio Grande do Sul Porto Alegre RS Brazil; ^2^ Faculdade de Farmácia Universidade Federal do Rio Grande do Sul Porto Alegre RS Brazil; ^3^ Laboratório de Síntese Orgânica Medicinal (LaSOM), Faculdade de Farmácia Universidade Federal do Rio Grande do Sul Porto Alegre RS Brazil

**Keywords:** chiral, enantioselectivity, green solvents, HPLC, nifurtimox

## Abstract

Nifurtimox (NFX) is a chiral drug used for the treatment of Chagas Disease. Little attention has been paid to the enantioselective properties of chiral drugs used for neglected tropical diseases, highlighting the need for further studies in this area. In this work, the enantioselective properties of NFX were carefully investigated by HPLC using different chiral stationary phases (CSPs) and chromatographic modes. Two enantioseparation HPLC methods were successfully developed and validated. Polar ionic, polar organic, and RP modes were tested, and the polar organic mode proved to be suitable for the enantioseparation. Chromatographic resolution was achieved using the polysaccharide‐based Chiralpak AD CSP and the macrocyclic glycopeptide‐based Chirobiotic V CSP. Both methods employed ethanol as a mobile phase, contributing to greener analytical practices. The influence of the CSPs temperature on the retention of the enantiomers was investigated, and the analysis temperatures were set at 25°C. Thermodynamic parameters were also studied, and the enantioseparation process was found to be enthalpy‐driven. Furthermore, molecular docking studies were conducted to identify the interactions of NFX with the CSPs constituents and to predict the elution order of the enantiomers. The elution order of the enantiomers was determined by comparing the experimental electronic circular dichroism (ECD) spectra with the theoretical ECD spectra, which aligned with the predictions made through molecular docking. The environmental impact of the developed methodologies was evaluated using the green metrics MoGAPI and AGREE, confirming their eco‐friendly nature. The methods can be employed in future enantioselectivity studies and drug analysis.

## 1 Introduction

Chagas disease, also known as American trypanosomiasis, is an infectious disease caused by the protozoan parasite *Trypanosoma cruzi*. About 6–7 million people worldwide are estimated to be infected with *T. cruzi*, mostly in Latin America. After an acute infection, around 30% of the patients develop life‐threatening clinical complications of varying severity, affecting the digestive, heart, and nervous systems. World Health Organization (WHO) recognized Chagas disease as a neglected tropical disease in 2005, as it is especially prevalent in tropical areas and is almost absent from the global health agenda. However, due to international migration, the disease has also been reported in non‐endemic regions such as North America, Europe, Japan, and Australia [1, 2, 3, 4].

**FIGURE 1:**
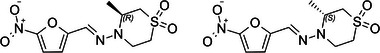
Structure of (R) and (S) enantiomers of NFX.

Only two drugs are currently available for treating Chagas disease, and both were developed more than 50 years ago: benznidazole and nifurtimox (NFX). Both drugs are associated with high levels of toxicity and side effects, including mutagenicity and genotoxicity. Also, the duration of the treatment is long (60–90 days) and has not been effective in chronic patients [5, 6, 7]. NFX has been supplied to the affected population through WHO special programs and, in 2020, was approved by the Food and Drug Administration (FDA) for use in pediatric patients. NFX is a 5‐nitrofuran derivative, it has the chemical name (RS)‐*N*‐(3‐methyl‐1,1‐dioxidothiomorpholin‐4‐yl)1‐(5‐nitro‐2‐furyl)methanimine, chemical formula C_10_H_13_N_3_O_5_S, and molecular weight of 287.29 g/mol. NFX has one chiral center (Figure 1) and is sold in the form of a racemate [8, 9, 10, 11].

It is well established that chiral drugs can present remarkable differences in pharmacology and pharmacokinetics, characterized expressly by therapeutic activity, inactivity, or even toxicity of the enantiomers [12]. However, only one study was found in the literature regarding the enantioselective properties of NFX, suggesting that the enantiomers would be equivalents [13]. Even a slight improvement in efficacy and a decrease in the side effects of an enantiomer could possibly contribute to NFX treatment adherence. Therefore, further complementary studies are encouraged, mainly because little attention has been paid to drugs for neglected tropical diseases. This creates a need to develop analytical methods capable of separating the enantiomers of NFX to be used in future enantioselectivity studies and application in drug quality control.

A widely used technique for separating and determining enantiomeric drugs is HPLC, recognized for its speed, selectivity, sensitivity, and high efficiency. The most common HPLC approach for separating enantiomers involves using chiral stationary phases (CSPs). Currently, various commercial CSP classes are available, such as polysaccharide, ligand‐exchange, Pirkle‐type, crown ether, cyclodextrin, and macrocyclic glycopeptide. The main chromatographic modes for chiral separation are normal‐phase, RP, polar ionic, and polar organic modes [14, 15, 16, 17, 18].

To further understand the chiral separation mechanisms and the order of enantiomer elution in chromatographic systems, computational studies have been increasingly applied [19]. One of the most popular studies is molecular docking, which consists of simulating possible connections of the drug (referred to as ligand) within the chiral selector (referred to as receptor) and estimating the goodness of this connection (referred to as docking score, often associated with binding affinity). Also, electronic circular dichroism (ECD) spectra can be theoretically calculated and compared to experimental results to attribute the absolute configuration of the eluted enantiomers [20].

To date, no methodology has been reported for the enantioseparation of NFX, and there has been no investigation into the elution order of the enantiomers. Chankvetadze and collaborators carried out in 2002 a comparative study of the enantioseparation of several chiral drugs using different CSPs of polysaccharides with polar organic mobile phases. NFX was one of the drugs studied; although the conditions tested provided adequate separation factors, the peak had low efficiency, and no baseline enantioseparation was achieved [21].

Nowadays, there is a great concern about environmental issues and eco‐friendly practices. As a result, the development of greener chiral analytical methodologies is essential in the field of pharmaceutical analysis [22, 23, 24, 25]. To support this effort, several green metrics have been developed to assess the degree of the environmental impact of analytical methodologies, being the more commonly used the Modified Green Analytical Procedure Index (MoGAPI) and the Analytical GREEnness Metric (AGREE) [26, 27, 28].

In view of the above, this work aims to investigate the enantioselective properties of NFX by HPLC with different CSP types, chromatographic modes and temperatures, meanwhile developing an HPLC method for the enantioseparation of NFX in tablets. In addition, it aims to implement greener analytical practices through the development of the methodologies and to use computational studies to understand and predict the elution order of the enantiomers.

## 2 Materials and Methods

### 2.1 Chemicals and Materials

NFX reference standard with an assigned purity of 98% was supplied by Sigma‐Aldrich, USA. Lampit tablets 120 mg (Bayer) for oral administration were donated by the Ministry of Health of Brazil. All chemicals and solvents were of either chromatographic or analytical grade. Methanol (MeOH), ACN, and triethylamine were obtained from Merck, Germany. Ethanol (EtOH) was obtained from Panreac, Spain. 2‐Propanol was obtained from Chromasolv, USA. Glacial acetic acid and ammonium acetate were obtained from Sigma‐Aldrich, USA. The excipients of the tablets were obtained from Synth, Brazil (calcium hydrogen phosphate dihydrate, magnesium stearate, maize starch, silica colloidal anhydrous, and sodium lauryl sulfate). Ultrapure water was produced using a Direct‐Q apparatus, Millipore, France.

The sample stock solution of 100 µg/mL of NFX was prepared by weighing an accurate amount of the tablet powder and diluting it in MeOH. The standard stock solution of 100 µg/mL of NFX was also prepared in MeOH. Working solutions were obtained by appropriate dilution of the stock solutions in MeOH to a concentration of 15 µg/mL. Since it is a racemic mixture, a concentration of 7.5 µg/mL of each enantiomer of NFX was obtained. The placebo sample was prepared containing all excipients present in the formulation of the NFX tablets. All the solutions were filtered through a 0.45 µm membrane filter, stored at 2°C–8°C, and protected from light.

### 2.2 Instrumentation and Conditions

HPLC analyses were performed using an Agilent 1200 series (Agilent, USA) equipped with a G1311A quaternary pump, ALS‐G1329A autosampler, TCC‐G1316A column oven, G1315B photodiode array detector, and ChemStation software for data processing and acquisition. The CSPs evaluated were: Chirobiotic T (150 × 4.6 mm, 5 µm) based on glycopeptide teicoplanin (Astec, USA); Chirobiotic V (150 × 4.6 mm, 5 µm) based on glycopeptide vancomycin (Astec, USA); Chiralpak AD (150 × 4.6 mm, 5 µm) based on amylose tris(3,5‐dimethylphenylcarbamate) (Daicel Chiral Technologies, China). The injection volume was 10 µL, and the detection wavelength was set at 395 nm.

The ECD spectra of enantiomeric fractions of NFX were recorded on a Jasco J‐815 spectrometer (Jasco, Japan) using a 10 mm path length cell at 25°C. Spectra were recorded from 210 to 800 nm at 1 nm intervals using a 1 nm spectral bandwidth, a 100 nm/min scanning speed, and a 2 s time constant. The ECD spectra were background corrected with EtOH. The enantiomeric fractions of NFX corresponding to the first‐eluted enantiomer (E1) and the second‐eluted enantiomer (E2) were collected using the developed method with the Chiralpak AD column. The concentrations were adjusted to keep the absorbance in the optimum photometric range.

### 2.3 Computational Studies

#### 2.3.1 Molecular Docking

The protocol started obtaining the SMILES notation of NFX enantiomers from Pubchem [29]. Then, the respective tridimensional structures were built from the SMILES in Wavefunction Spartan ‘14 v.1.1.8 [30], on which the molecules had their geometry optimized through molecular mechanics followed by semiempirical methods (MMFF + PM6). The chemical constituents’ tridimensional structures from Chirobiotic T and Chirobiotic V, teicoplanin and vancomycin, were obtained from the Protein Data Bank (PDB) using the PDBID codes 3mgb and 1aa5, respectively. Chiralpak AD structure was obtained from Ye and collaborators [31]. All the structures were preprocessed to include hydrogen atoms using the dockprep module of UCSF Chimera [32]. The chain C from the structure containing teicoplanin (PDBID: 3mgb) was selected, whereas the other atoms were removed. The vancomycin containing structure (PDBID: 1aa5) corresponding to chains A, B, and C was trimmed and united in a single structure containing only one conformation. The molecular docking was accomplished without solvent effects (vacuum conditions) in CCDC GOLD 5.2 [33]. The protocol developed in GOLD used the following parameters: (a) number of genetic algorithm runs = 10; (b) scoring function = ChemPLP; (c) genetic algorithm settings = default. The grid locations and sizes utilized in docking are reported in Table S1. The interaction profile was performed in Discovery Studio Visualizer 2025 [34] and the figures were obtained in Pymol 3.0 [35].

#### 2.3.2 ECD Spectra Simulation

The previously optimized structures from (R)‐NFX enantiomer were used as a starting point for conformational search carried out with MMFF force field in Spartan ‘14 [30]. The maximum number of conformers examined was set as 10 000. The Density Functional Theory (DFT) and simplified Time‐Dependent Density Functional Theory (sTD‐DFT) calculations were performed in Orca 6.0.1 [36, 37]. The resulting conformers obtained from Spartan ‘14 were converted in “.xyz” extension. Then, their geometries were further refined using B3LYP functional and D4 dispersion correction (B3LYP‐D4) employing def2‐SVP as basis set and considering a Conductor‐like Polarizable Continuum Model (CPCM) of EtOH as implicit solvent. In the next step, the frequencies were obtained using the B3LYP‐D4/def2‐TZVP and CPCM model of EtOH with TightSCF convergence criteria. Next, the first 80 excited states were calculated via sTD‐DFT method [38] at ωB97X‐D3/def2‐TZVP, CPCM model of EtOH and TightSCF convergence criteria. The calculated ECD data were analyzed and processed in SpecDis tool 1.71 [39], in which the enantiomers spectra were obtained according to the Boltzmann distribution of the most representative conformations and compared to the experimental data. The obtained spectra were shifted 10 nm and the sigma was set in 0.22 eV. Further figure enhancements were achieved in Inkscape 1.3.

### 2.4 Optimized Final Methods

Two methodologies were successfully developed for the enantioseparation of NFX. The chromatographic conditions with the Chirobiotic V column were: mobile phase of EtOH, flow rate of 0.4 mL/min, column oven temperature at 25°C, injection volume of 10 µL, and detection wavelength of 395 nm. The chromatographic conditions with the Chiralpak AD column were mobile phase of EtOH, flow rate of 1 mL/min, column oven temperature at 25°C, injection volume of 10 µL, and detection wavelength of 395 nm. Chromatographic analyses were performed in triplicate.

### 2.5 Method Validation

The developed methodologies were validated according to official guidelines in terms of selectivity, linearity, LOD, LOQ, precision (repeatability and intermediate precision), accuracy, and robustness [40].

The selectivity was determined by comparative analysis between NFX and placebo samples. The absence of possible interference was assessed by the peak purity of the enantiomers using the ChemStation software tool.

The linearity was evaluated through the construction of three independent standard calibration curves on three different days, with five concentration levels in the range of 5–25 µg/mL of NFX reference standard. The enantiomer's peak areas were measured and plotted against the corresponding concentration levels to determine the regression equation and correlation coefficients. Linear regression and deviation from linearity were determined by analysis of variance (ANOVA). Also, the homoscedasticity was assessed by the Cochran *C*‐test. LOD and LOQ were established based on the SD of the linear response and the slope of the standard calibration curve.

The precision was evaluated by repeatability and intermediate precision and expressed by relative SD (RSD%). The repeatability was evaluated by analyzing six independent samples of NFX (15 µg/mL) on the same day. The intermediate precision was assessed by analyzing six independent samples of NFX (15 µg/mL) on two consecutive days.

The accuracy was verified by recovering known amounts of NFX reference standard added to a placebo sample, obtaining three concentration levels (low, medium, and high) of each enantiomer. The added concentrations were 5, 15, and 25 µg/mL, which were prepared in triplicate. The results were evaluated as the mean percent recovery of the theoretical value compared to the experimental value.

Robustness was evaluated by performing deliberate variations in the chromatographic conditions using a Plackett–Burman experimental design. The factors studied were flow rate (± 0.1 mL/min), detection wavelength (± 1 nm), and column oven temperature (± 0.5°C), while the response evaluated was the chiral resolution. The results were statistically examined by ANOVA and graphically interpreted by Pareto charts.

## 3 Results and Discussion

### 3.1 Chiral Separation of NFX in Polar Ionic and RP Modes

The polar ionic and RP modes were investigated with the CSPs Chirobiotic V and T. In the polar ionic mode, the initial mobile phase consisted in MeOH:acetic acid:triethylamine (100:0.1:0.1, v:v:v). Then, acid‐base ratio changes, ammonium acetate addition, and ACN and EtOH solvents were also tested. In the RP mode, percentage and type of organic solvent (MeOH, ACN, and EtOH), buffer type and concentration (ammonium acetate and triethylamine acetate), and adjustment of pH (4–6) were evaluated [41, 42, 43, 44]. Despite the different conditions tested, all changes resulted in the same co‐elution chromatographic profile. The result can be explained by the fact that the NFX is not easily ionized, and therefore, no ionic interactions with the CSPs occurred. Furthermore, it appears that hydrophobic interactions were also not strong. Consequently, the organic polar mode was chosen for further studies.

### 3.2 Chiral Separation of NFX in Polar Organic Mode

The polar organic mode was investigated with the CSPs Chirobiotic V, Chirobiotic T, and Chiralpak AD. The organic solvents evaluated as mobile phase were ACN, MeOH, and EtOH, at a flow rate of 0.5 mL/min [45].

**FIGURE 2:**
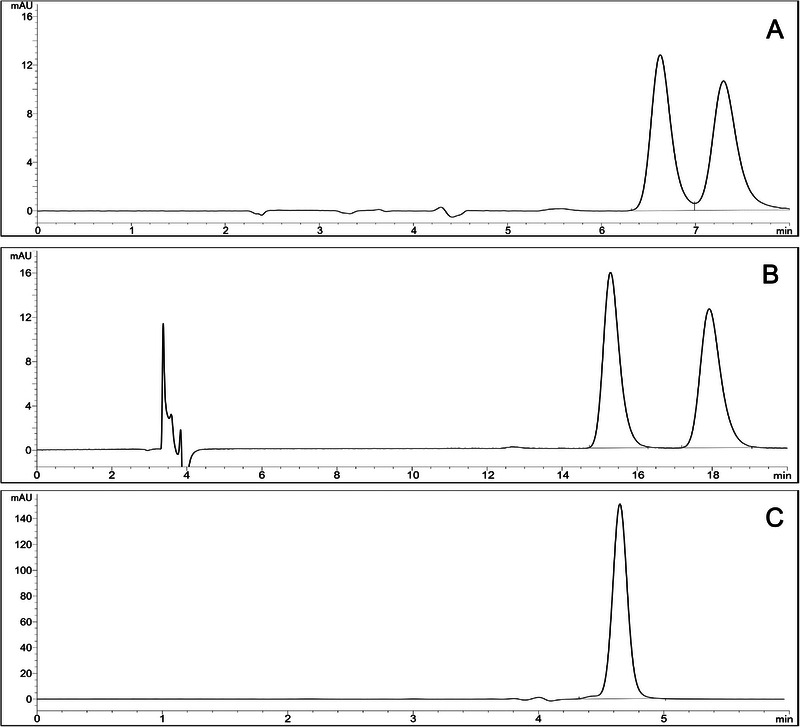
Comparison of enantioseparation behavior of NFX (15 µg/mL) in polar organic mode in different columns: (A) Chirobiotic V, (B) Chiralpak AD, and (C) Chirobiotic T. Chromatographic conditions: mobile phase EtOH, flow rate of 0.5 mL/min, column oven temperature at 25°C, injection volume of 10 µL, and detection wavelength of 395 nm.

With the Chirobiotic V, only EtOH provided the enantioseparation of NFX, with adequate resolution, run time, retention factor (*k*), selectivity factor (*α*), and plates, and therefore selected as the mobile phase for this CSP (Figure 2). With the Chirobiotic T, no enantioseparation was obtained. Although the differences between Chirobiotic V and T are subtle (number of inclusion pockets and interactive site types and distances), only Chirobiotic V was able to separate NFX enantiomers [46]. In 2023, Andersson and collaborators reported that Chirobiotic V was more suitable for separating a group of neutral compounds than Chirobiotic T, corroborating the results found [47].

In the Chiralpak AD, all solvents successfully separated the NFX enantiomers at an initial flow rate of 0.5 mL/min. ACN had a short retention time of the enantiomers, close to 5 min. It provided an adequate resolution, but the values obtained for *k* (< 1.0) and plates (< 2000) demonstrate that the separation was not efficient. MeOH and EtOH had similar results, with adequate resolution, *k*, *α*, and plates, but run times close to 20 min.

In the Green Analytical Chemistry context, less toxic solvents should be used whenever possible without compromising analytical performance. ACN and MeOH present some issues in terms of environmental impact and health safety. Both are considered toxic solvents and have significant requirements for waste disposal. EtOH, on the other hand, is considered a green organic solvent [48]. Therefore, aiming to develop more environmentally friendly and sustainable methodologies and considering the similar results obtained, EtOH was chosen as the mobile phase over MeOH with the Chiralpak AD column (Figure 2).

#### 3.2.1 Effect of Flow Rate

Following the establishment of the use of Chirobiotic V and Chiralpak AD CSPs with EtOH as the mobile phase, the effect of flow rate was studied. With the Chirobiotic V, the initial flow rate of 0.5 mL/min provided a run time shorter than 10 min. Optimization in the range of 0.3–0.6 mL/min showed inadequate values of resolution above the flow rate of 0.5 mL/min. The best compromise among retention time, resolution, and plates was achieved in the flow rate of 0.4 mL/min, which was set as the final flow rate (Table S2). With the Chiralpak AD, a high run time close to 20 min was obtained at a flow rate of 0.5 mL/min. Increasing the flow rate to the manufacturer's recommended maximum of 1 mL/min reduced the run time to under 10 min while maintaining adequate resolution, *k*, *α*, and plates. Thus, the flow rate with the Chiralpak AD was set to 1 mL/min.

#### 3.2.2 Effect of Temperature and Thermodynamic Parameters

It is well established that temperature can affect the intermolecular interactions responsible for analyte retention and enantioselectivity. At higher temperatures, peak efficiency usually improves at the expense of some decrease in enantioselectivity. On the other hand, lowering the temperature can attenuate weaker interactions, leading to better enantioseparation. Therefore, investigating the influence of temperature and evaluating the thermodynamic parameters of the enantiomers can provide valuable insights into the chiral separation mechanisms [41, 44].

**FIGURE 3:**
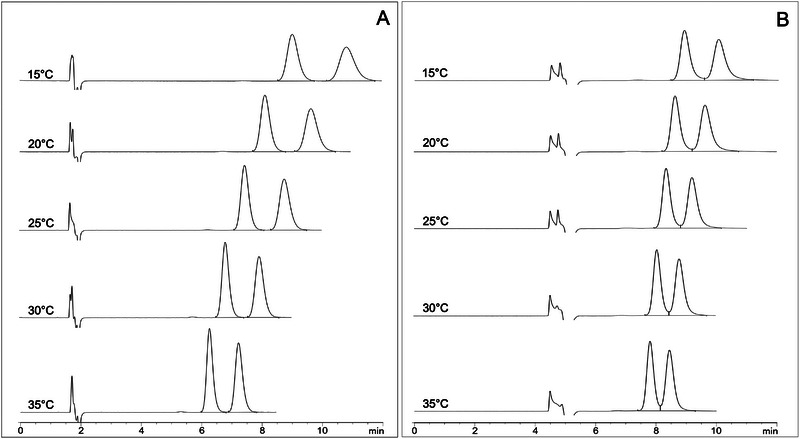
Illustrative chromatograms of effect of temperature on enantioseparation and resolution of NFX in: (A) Chiralpak AD and (B) Chirobiotic V. Chromatographic conditions: mobile phase EtOH, flow rate of 1.0 mL/min for Chiralpak AD and 0.4 mL/min for Chirobiotic V, injection volume of 10 µL, and detection wavelength of 395 nm.

In order to acquire information about the chiral recognition mechanism between NFX and the Chiralpak AD and Chirobiotic V columns, the effect of the temperature was studied in the range of 15°C–35°C. The experimental data obtained are summarized in Table 1, and the illustrative chromatograms are shown in Figure 3. In both columns, it was observed that as the temperature increased, the values of *k* and *α* decreased. However, higher temperatures provided shorter retention time, improved column efficiency, and sharper peaks. With the Chirobiotic V column, the resolution of the enantiomers decreased with increasing temperature. Interestingly, for the Chiralpak AD column, the highest resolution was obtained at the intermediate temperature of 25°C. Taking these factors into consideration and aiming to facilitate the operational temperature control, 25°C was selected as the optimum temperature for NFX enantioseparation in both CSPs.

**TABLE 1:** The effect of column oven temperature on retention and enantioseparation of NFX.

Temperature	Chiralpak AD				Chirobiotic V			
	Rs	*α*	*k*		Rs	*α*	*k*	
			E1	E2			E1	E2
15°C	2.23	1.24	5.03	6.23	1.82	1.22	1.38	1.69
20°C	2.34	1.23	4.43	5.45	1.74	1.20	1.30	1.57
25°C	2.37	1.22	3.96	4.84	1.63	1.19	1.22	1.44
30°C	2.37	1.21	3.56	4.30	1.52	1.17	1.14	1.34
35°C	2.33	1.20	3.19	3.83	1.40	1.16	1.07	1.24

*Note*: Chromatographic conditions: mobile phase EtOH, flow rate of 1.0 mL/min for Chiralpak AD and 0.4 mL/min for Chirobiotic V, injection volume of 10 µL, and detection wavelength of 395 nm.

Abbreviations: Rs, resolution; *α*, selectivity factor; *k*, retention factor; E1, first‐eluted enantiomer; E2, second‐eluted enantiomer.

To evaluate the enthalpic and entropic contributions of enantioselective adsorption of both columns, Van't Hoff plots were constructed, which may be interpreted in terms of the mechanistic of chiral recognition [44, 49], based on Equation 1:

(1) $$\ln\;k=-\frac{\Delta H_{i}^{\circ }}{RT}+\frac{\Delta S_{i}^{\circ }}{R}+\ln\;\phi \#$$

where *k* is the retention factor, Δ*H*° and Δ*S*° are the standard molar enthalpy and entropy, respectively, of transfer of the enantiomer from the mobile phase to the stationary phase, *R* is the universal gas constant, *T* is the absolute temperature, *ϕ* is the phase ratio of the chromatographic column, and subscript *i* denotes the corresponding species (e.g., here (R) and (S) enantiomers). Since the value of *ϕ* is not always known, the Δ*S*° values are generally reported as Δ*S*°* without considering *ϕ*.

Similarly, Equation 2 expresses the difference between enthalpy variation (ΔΔ*H*°) and standard entropy variation (ΔΔ*S*°) for each enantiomer, where *α* is the selectivity factor:

(2) $$\ln\;\alpha =-\frac{\Delta \left(\Delta H^{\circ }\right)}{RT}+\frac{\Delta \left(\Delta S^{\circ }\right)}{R}\#$$

These equations reveal that if the plots of the natural logarithm of *k* versus 1/*T* and the natural logarithm of *α* versus 1/*T* are linear, then the slope can be assumed as −Δ*H*°/*RT* and the intercept as Δ*S*°/*R*, which are invariant with temperature [44, 49].

The plots of ln *k* versus 1/*T* were constructed from experimental chromatographic data (Table 1) and demonstrated to be linear (*r* ^2^ > 0.999) (Figure S1). The Δ*H*° and Δ*S*°* values calculated from the slopes and intercepts of the plots were all negative. This suggests that there is no change in the separation mechanism across the temperatures studied. Also, the E2 in both columns showed more negative Δ*H*° and Δ*S*° values, indicating a stronger interaction with the CSP [50, 51]. Likewise, the plots of ln *α* versus 1/*T* demonstrated to be linear (*r* ^2^ > 0.999) (Figure S1). The ΔΔ*H*° and ΔΔ*S*° were negative in both columns, indicating that the enantioseparation process is enthalpy‐driven, as typically reported [52, 53, 54].

Moreover, the isoenantioselective temperatures (*T*_iso_) were determined as the ratio between ΔΔ*H*° and ΔΔ*S*°. *T*_iso_ represents the point at which the enthalpy and entropy compensate each other, resulting in the co‐elution of the two enantiomers. Therefore, above the *T*_iso_, the elution order of the enantiomer's reverses compared to below it. In the NFX separation, the *T*_iso_ was found to be 149°C and 115°C for the Chiralpak AD and Chirobiotic V columns, respectively. This indicates that there will be no change in the elution order of the enantiomers within the temperature range of 15°C–35°C on both columns [50, 55, 56].

It is worth mentioning that the thermodynamics parameter values determined from analytical chromatography data include achiral components and are not exclusively due to enantioselective analyte retention. Also, a comprehensive investigation requires accurate *k* measurements, including *ϕ* in the equation, considering involved partial processes (net adsorption, desolvation, desorption, resolvation, and dilution), etc. Nevertheless, these approximated values still offer insights into the chiral separation mechanisms [49, 57, 58]. All the calculated thermodynamic parameters on both CSPs are presented in Table S3.

### 3.3 Computational Studies

#### 3.3.1 Molecular Docking

Molecular docking studies of NFX enantiomers were establish on each CSP structure to predict the drug interaction profile as well as the elution order. The data obtained in GOLD using ChemPLP scoring function predicted S‐NFX as the first enantiomer to be eluted in all the three CSPs evaluated (Table 2).

**TABLE 2:** Docking scores and the predicted elution order of NFX according to the CSPs.

Chirobiotic V		
Compound	ChemPLP (GOLD)	Elution order (GOLD)
R‐NFX	46.6739	**S followed by R**
S‐NFX	45.6511	
**Chirobiotic T**		
Compound	ChemPLP (GOLD)	Elution order (GOLD)
R‐NFX	55.0444	**S followed by R**
S‐NFX	47.5329	
**Chiralpak AD**		
Compound	ChemPLP (GOLD)	Elution order (GOLD)
R‐NFX	36.4437	**S followed by R**
S‐NFX	32.6098	

**FIGURE 4:**
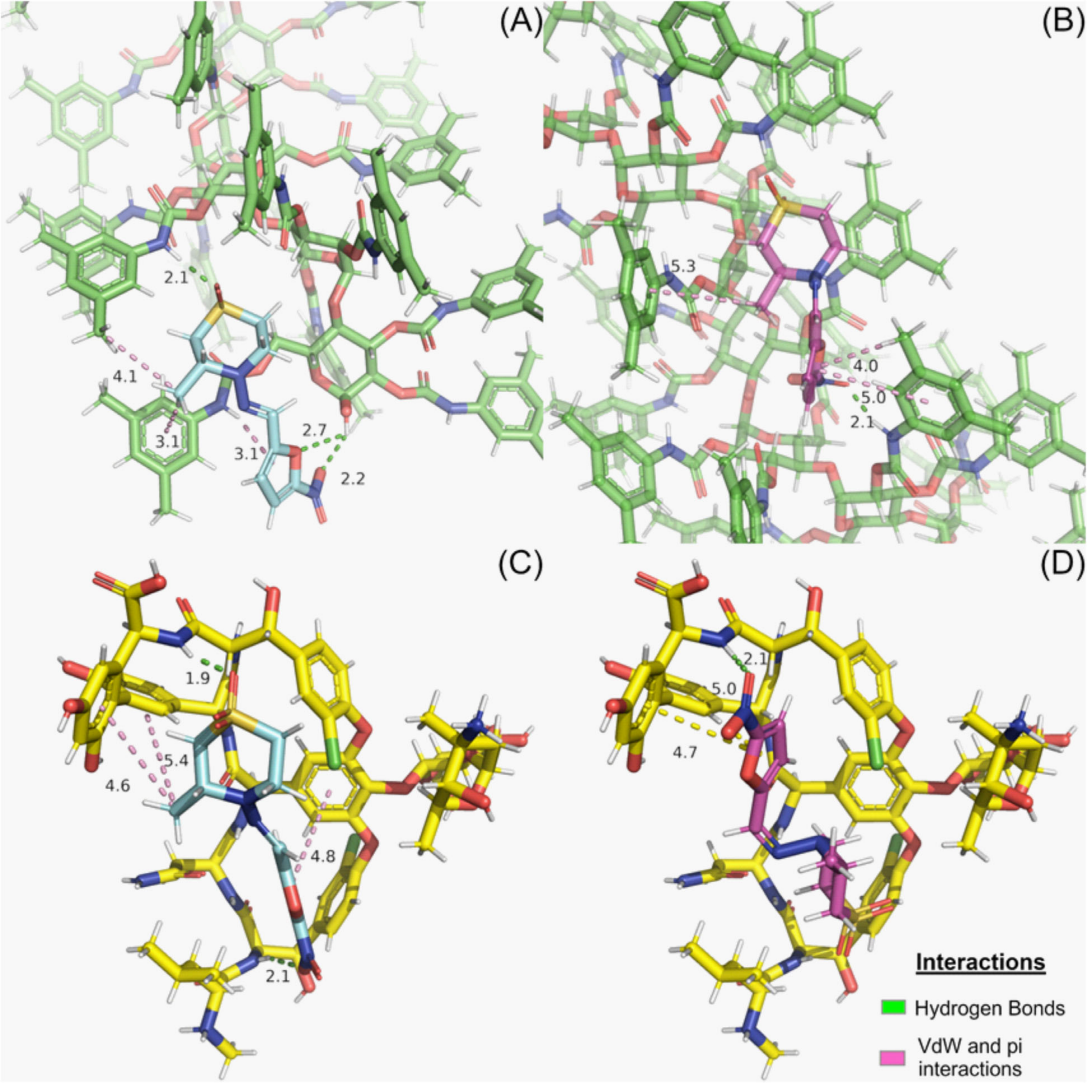
Interaction profile obtained for the best poses of NFX enantiomers in Chiralpak AD and in Chirobiotic V: (A) (R)‐Nifurtimox in Chiralpak AD, (B) (S)‐Nifurtimox in Chiralpak AD, (C) (R)‐Nifurtimox in Chirobiotic V, and (D) (S)‐Nifurtimox in Chirobiotic V. Chiralpak AD CSP is represented with green carbon sticks, Chirobiotic V is represented with yellow carbon sticks, (R)‐Nifurtimox enantiomer is represented in cyan carbon sticks, and (S)‐Nifurtimox enantiomer is represented in magenta carbon sticks. Hydrogen bond interactions are represented in green dashed lines, whereas van der Waals and pi‐interactions are represented in pink dashed lines.

The interaction profile analyses showed that NFX was able to perform hydrogen bonds, van der Waals, and pi‐interactions (𝝿‐stacking and 𝝿‐alkyl) with the CSPs. The hydrogen bonds were accomplished by NFX's nitro group acting as acceptor with NH of Chiralpak AD, Chirobiotic V, and Chirobiotic T and to lesser extension by NFX's sulfone group, which also act as acceptor with NH of Chiralpak AD and Chirobiotic V (Figure 4). Ionic interactions of NFX with CSPs were not observed as expected. NFX assumed different poses considering the enantiomer in Chiralpak AD and in Chirobiotic V. Nonetheless, the drug assumed a similar pattern of poses regarding the 1,1‐dioxo‐1,4‐thiazinan‐4‐yl moiety in Chirobiotic T (Figure S1), mainly differing in the nitrofuran group. This pattern may explain the low enantiodiscrimination power experimentally observed for Chirobiotic T. Phyo and collaborators conducted a related work consisting of molecular docking of several xanthonic derivatives on chirobiotic columns employing Autodock Vina. The authors observed similar interaction types (e.g., hydrophobic, 𝝿‐stacking, and hydrogen bonds) [59].

Nevertheless, molecular docking exhibits certain limitations, particularly regarding the accurate modeling of solvent effects. The method is routinely employed to predict the binding modes and affinities of ligands within the active sites of target receptors, typically proteins. Although some docking algorithms allow the inclusion of solvent‐related parameters or utilize implicit solvation models, the explicit representation of solvent molecules remains a significant challenge, as they are often excluded from the docking system during the preparation stage.

#### 3.3.2 ECD Spectra Simulation

The elution order of the enantiomers was determined by calculating the theoretical ECD spectra for the (R)‐NFX and comparing it with the experimental ECD spectra measures of the eluted peaks; obtained by the micro‐preparative enantioseparation described in Section 2.2.

**FIGURE 5:**
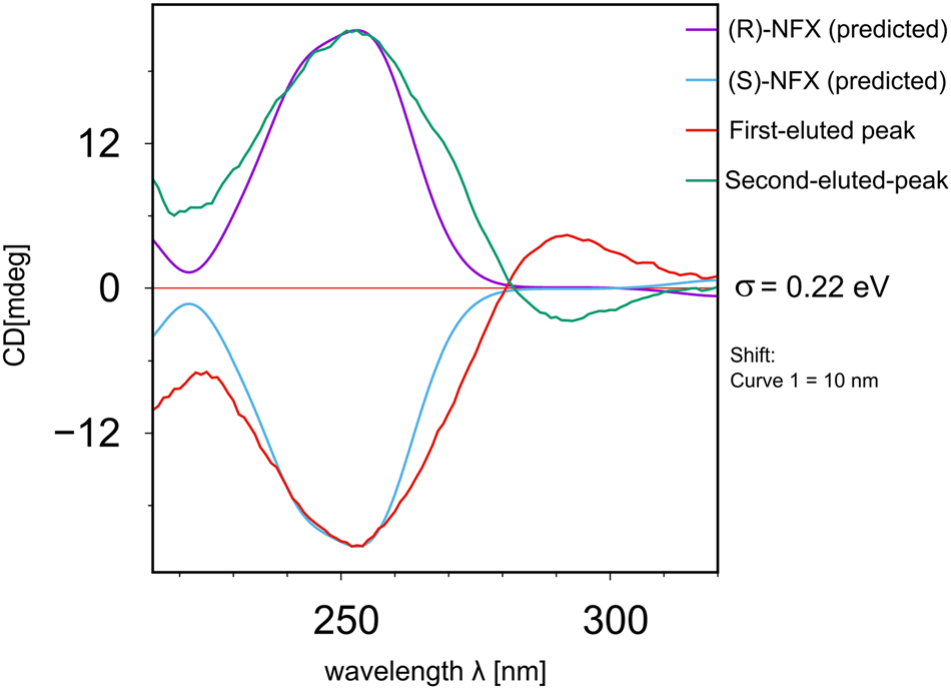
Comparison of predicted and experimental ECD spectra of NFX. The predicted spectra of (R)‐NFX and (S)‐NFX are represented in purple and light blue curves, respectively. The experimental spectra of the first‐eluted peak and second‐eluted peak are represented in red and green curves, respectively. The predicted spectra were shifted 10 nm for fitting.

The theoretical ECD spectra were simulated for the eight conformations of (R)‐NFX through sTD‐DFT approach considering 80 excited states. The conformers obtained by DFT optimization did not report any imaginary frequencies, which could indicate the presence of a high energy conformation (i.e., transition‐states). The most representative conformers were used to draw a Boltzmann‐weighted spectrum and compared to experimental data. The obtained spectrum for (R)‐NFX was predicted as positive regarding the employed wavelength range, whereas the (S)‐NFX was obtained reflecting the original spectrum (Figure 5). We may observe that the predicted spectra superposed the experimental findings. Both experimental and simulated spectra displayed maximum and minimum energy values at 250 nm. The comparison of the predicted and experimental ECD spectra allowed the inference of the absolute configuration (R or S enantiomer) to the first‐ and second‐eluted compounds. The E1 was found to be the (S)‐enantiomer and the E2 the (R)‐enantiomer, in both methodologies. Assuming this correspondence, we may further analyze the docking results regarding the elution order prediction. The docking poses and scores reported by GOLD matched the elution order observed experimentally.

### 3.4 Validation

**FIGURE 6:**
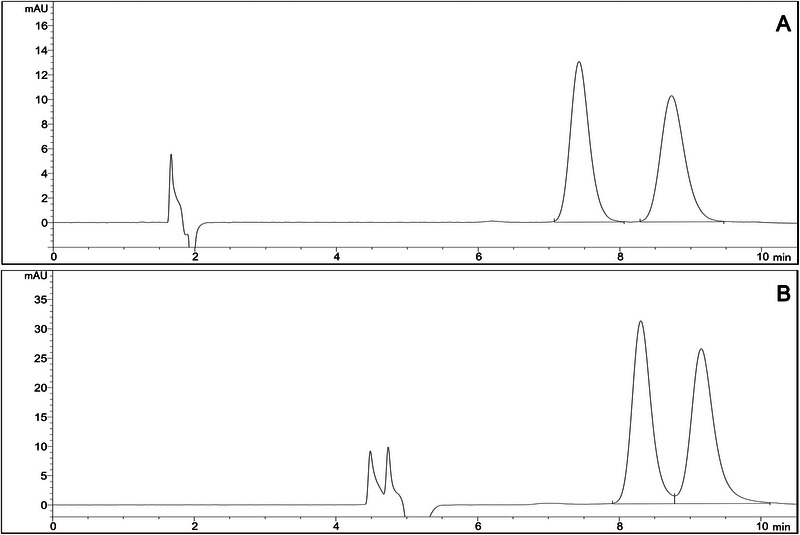
Illustrative chromatogram of the two developed methodologies for the enantioseparation of NFX: (A) Method in Chiralpak AD CSP and (B) Method in Chirobiotic V CSP. Chromatographic conditions: mobile phase EtOH, flow rate of 1.0 mL/min for Chiralpak AD and 0.4 mL/min for Chirobiotic V, injection volume of 10 µL, and detection wavelength of 395 nm.

Method validation is crucial for confirming that the proposed methods are adequate and reliable for their intended purpose. Figure 6 illustrates the chromatograms obtained in the final conditions of the two developed methodologies to separate the enantiomers of NFX. The first methodology conditions were: Chiralpak AD CSP, mobile phase of EtOH, flow rate of 1 mL/min, and column oven temperature at 25°C. For the second methodology, the optimum conditions were: Chirobiotic V CSP, mobile phase of EtOH, flow rate of 0.4 mL/min, and column oven temperature set at 25°C. The values obtained in the system suitability test are described in Table 3.

**TABLE 3:** System suitability test of optimized chromatographic conditions of the two developed methodologies.

		E1			E2		
CSP	Rs	Rt (min)	Plates	*k*	Rt (min)	Plates	*k*
Chiralpak AD	2.37	7.42	3767	3.96	8.72	3193	4.84
Chirobiotic V	1.63	8.29	4685	1.22	9.14	4281	1.44

*Note*: Acceptable ranges considered: Rs > 1.5; Plates > 2000; 1 < *k* < 5. Analyses were performed in triplicate.

Abbreviations: Rs, resolution; Rt, retention time (min); *k*, retention factor; E1, first‐eluted enantiomer; E2, second‐eluted enantiomer.

Both methodologies were validated according to Section 2.5. The excipients in the tablet formulation did not interfere with the determination of the NFX enantiomers (Figure S3), indicating the selectivity of the methodologies. For the linearity, statistical analysis by ANOVA showed linear regression and no deviation from linearity of the enantiomers on both methodologies. The analysis of the regression residues showed random dispersion with the absence of outliers, and the homoscedasticity was confirmed. Range, slope, intercept, and correlation coefficient of the calibration curves, as well as the LOD and LOQ, are provided in Table 4.

**TABLE 4:** Validation data of linearity, LOD, LOQ, precision, and accuracy of both developed methodologies for the determination of NFX enantiomers.

	Chiralpak AD		Chirobiotic V	
	E1	E2	E1	E2
Linearity				
Range (µg/mL)	5–25	5–25	5–25	5–25
Slope	19.08 (±0.31)	19.00 (±0.30)	47.83 (±0.55)	49.26 (±0.40)
Intercept	−2.99 (±5.17)	−3.46 (±5.08)	−15.43 (±9.12)	−30.33 (±6.64)
Correlation coefficient	0.9982	0.9983	0.9991	0.9995
**LOD (µg/mL)**	0.89	0.88	0.62	0.44
**LOQ (µg/mL)**	2.70	2.67	1.90	1.34
**Precision (RSD%)**				
Repeatability (*n* = 6)	1.78	1.83	1.71	1.61
Intermediate precision (*n* = 12)	1.96	1.89	1.92	1.86
**Accuracy (% of recovery)**				
Low level	103.17	103.28	102.00	102.22
Medium level	98.98	98.75	101.68	102.87
High level	104.30	104.26	104.34	104.18

*Note*: All analyses were performed in triplicate.

Abbreviations: E1, first‐eluted enantiomer; E2, second‐eluted enantiomer; (± standard deviation).

The RSD of repeatability and intermediate precision were below 2% for both enantiomers with the Chirobiotic V and the Chiralpak AD column, indicating the precision of the analytical methods (Table 4). The percentage of recovery of the enantiomers of both methodologies was adequate, as presented in Table 4, indicating the accuracy of the methodologies. In the robustness assessment, statistical analysis showed that the deliberate variations in the chromatographic conditions did not significantly influence the chiral resolution of NFX enantiomers in both methodologies. These results indicate that the developed chiral HPLC methodologies for the determination of NFX enantiomers are selective, linear, sensitive, precise, accurate, and robust.

### 3.5 Green Assessment of the Developed Methods

The environmental impacts of the developed methodologies were evaluated using the green metrics MoGAPI and AGREE [23, 24, 25]. The overall scores for both methodologies were similar and higher than 0.74 (Figure S4). The combined results from MoGAPI and AGREE confirm the eco‐friendly nature of the developed methodologies, which can then be considered green chromatographic methods.

## 4 Conclusion

The enantioselective properties of NFX were explored using different CSPs, chromatographic modes and temperatures. Results showed important differences among the CSPs evaluated. The Chiralpak AD and the Chirobiotic V columns effectively separated NFX enantiomers, while the Chirobiotic T column did not demonstrate enantiodiscrimination power. Among the chromatographic modes, the polar organic mode was suitable for the enantioseparation due to the drug's characteristics. Two chiral methods were successfully developed for the determination of NFX enantiomers in tablets. EtOH was employed as the mobile phase in both methods, contributing to more environmentally friendly practices. In addition, the metrics MoGAPI and AGREE were applied to assess the environmental impact of the methods, confirming that they can be considered as green. The effect of temperature and thermodynamic parameters was also evaluated. In both columns, the enantioseparation process was enthalpy‐driven, and there was no change in the elution order of the enantiomers within the studied temperature range. The molecular docking studies conducted in GOLD predicted the S‐NFX as E1 in both CSPs. The ECD spectra simulation using the sTD‐DFT approach was able to match the NFX enantiomers as follows: E1 as (S)‐NFX and E2 as (R)‐NFX. Therefore, the elution order predicted by GOLD was consistent with the ECD experiments. Nonetheless, further molecular studies, including molecular dynamics, are encouraged to be conducted to predict the influence of solvents and energy on the molecular systems. The two developed methods were validated, proving to be selective, linear, sensitive, precise, accurate, and robust. This study contributes to understanding NFX enantioselectivity, and the methodologies developed can be applied in future studies on enantioselectivity and drug analysis.

## Author Contributions

**Amanda Mohr**: formal analysis, investigation, writing – original draft. **Érika Segala**: investigation. **Gustavo Machado das Neves**: investigation, writing – original draft. **Vera Lucia Eifler‐Lima**: writing – review and editing. **Martin Steppe**: conceptualization, resources, writing – review and editing, supervision.

## Conflicts of Interest

The authors declare no conflicts of interest.

## Supporting information




**Supporting File 1**: jssc70258‐sup‐0001‐SupMat.docx.

## Data Availability

Data are available on request from the authors.
