# Supplementary material for: Enantioselective Properties of Neglected Drug Nifurtimox on Polysaccharide and Macrocyclic Glycopeptide Chiral Stationary Phases by Green HPLC Separation Methods
Source: J Sep Sci. 2025 Sep 8;48(9):e70258. doi: 10.1002/jssc.70258 (PMC12417557; doi:10.1002/jssc.70258)
Supplement: Supplementary file 1 — Supporting File 1: jssc70258‐sup‐0001‐SupMat.docx. [file JSSC-48-e70258-s001.docx]

**Supplementary Material**

Enantioselective properties of neglected drug Nifurtimox on polysaccharide and macrocyclic glycopeptide chiral stationary phases

Amanda Mohr^a^*, Érika Segala*^b^*, Gustavo Machado das Neves*^c^*, Vera Lucia Eifler-Lima*^c^*, Martin Steppe^a^

*^a^Programa de Pós-Graduação em Ciências Farmacêuticas, Universidade Federal do Rio Grande do Sul, Av. Ipiranga 2752, Porto Alegre, RS, Brasil*

*^b^Faculdade de Farmácia, Universidade Federal do Rio Grande do Sul, Av. Ipiranga 2752, Porto Alegre, RS, Brasil*

*^c^Laboratório de Síntese Orgânica Medicinal (LaSOM), Faculdade de Farmácia, Universidade Federal do Rio Grande do Sul, Av. Ipiranga 2752, Porto Alegre, RS, Brasil*

*Correspondence author e-mail: amandamohr21@hotmail.com

**Table of contents**

**Table S1** - Molecular docking binding site parameters according to the CSP structure.

**Table S2** - Effect of flow rate on enantioseparation and retention of NFX (15 µg/mL) in Chirobiotic^®^ V CSP.

**Table S3** - Thermodynamic parameters in different CSP of NFX enantiomers.

**Figure S1** - Van’t Hoff plots for the separation of NFX enantiomers in (A) Chiralpak^®^ AD CSP and (B) Chirobiotic V^®^ CSP. E1 = first-eluted enantiomer; E2 = second-eluted enantiomer.

**Figure S2** - Interaction profile obtained for the best poses of NFX enantiomers in Chirobiotic^®^ T: (A) (R)-Nifurtimox; (B) (S)-Nifurtimox. Chirobiotic^®^ T CSP is represented with salmon carbon sticks, (R)-Nifurtimox enantiomer is represented in cyan carbon sticks and (S)-Nifurtimox enantiomer is represented in magenta carbon sticks. Hydrogen bond interactions are represented in green dashed lines, van der Waals and pi-interactions are represented in pink dashed lines and pi-sulfur interactions in yellow dashed lines.

**Figure S3** - Illustrative chromatograms demonstrating the selectivity of the methodologies: (A) Chiralpak^®^ AD, NFX sample solution, (B) Chiralpak^®^ AD, placebo solution, (C) Chirobiotic V^®^, NFX sample solution (D) Chirobiotic V^®^, placebo solution.

**Figure S4** - Environmental impact assessment of the developed methodologies: (A) MoGAPI pictogram of method in Chirobiotic^®^ V column, (B) MoGAPI pictogram of method in Chiralpak^®^ AD column, (C) AGREE pictogram of method in Chirobiotic^®^ V column, (D) AGREE method in Chiralpak^®^ AD column.

**Table S1** - Molecular docking binding site parameters according to the CSP structure.

| **CSP structure** | **Grid Location (XYZ)** | **CCDC GOLD**  **Grid radius (Å)** |
| --- | --- | --- |
| Chirobiotic^®^ V | 3.159258 8.632966 1.802899 | 10 |
| Chirobiotic^®^ T | -24.763388 -44.127322 -13.823237 | 10 |
| Chiralpak^®^ AD | 19.978451 20.073423 26.036014 | 30 |

**Table S2** - Effect of flow rate on enantioseparation and retention of NFX (15 µg/mL) in Chirobiotic^®^ V CSP.

| **Flow rate (mL/min)** | **Rs** | **α** | **Rt  (min)** | | | **k** | | | **Plates** | |
| --- | --- | --- | --- | --- | --- | --- | --- | --- | --- | --- |
|  |  |  | **E1** | **E2** | **E1** | | **E2** | **E1** | | **E2** |
| 0.3 | 1.69 | 1.19 | 10.96 | 12.07 | 1.20 | | 1.42 | 5232 | | 4653 |
| 0.4 | 1.61 | 1.19 | 8.17 | 9.01 | 1.19 | | 1.41 | 4723 | | 4131 |
| 0.5 | 1.54 | 1.19 | 6.55 | 7.22 | 1.19 | | 1.41 | 4293 | | 3870 |
| 0.6 | 1.48 | 1.19 | 5.46 | 6.02 | 1.19 | | 1.42 | 4006 | | 3621 |

**Notes:** Chromatographic conditions: mobile phase EtOH, column oven temperature at 25°C, injection volume of 10 µL, detection wavelength of 395 nm. Rs = resolution; α = selectivity factor; Rt = retention time (min); k = retention factor; E1 = first-eluted enantiomer, E2 = second-eluted enantiomer.

**Table S3** - Thermodynamic parameters in different CSP of NFX enantiomers.

| **CSP** | **ΔH° E1**  **(J × mol^−1^)** | **ΔH° E2**  **(J × mol^−1^)** | **ΔS°* E1**  **(J × K^−1^ × mol^−1^)** | **ΔS°* E2**  **(J × K^−1^ × mol^−1^)** | **ΔΔH°**  **(J × mol^−1^)** | **ΔΔS°**  **(J × K^−1^ × mol^−1^)** | **T*_iso_***  **(°C)** |
| --- | --- | --- | --- | --- | --- | --- | --- |
| Chiralpak^®^ AD | - 4994.07 | - 5332.53 | - 44.75 | - 46.91 | - 360.51 | - 2.41 | 149.72 |
| Chirobiotic^®^ V | - 2807.99 | - 3389.26 | - 29.97 | - 35.09 | - 555.50 | - 4.83 | 115.11 |

**Notes:** E1 = first-eluted enantiomer, E2 = second-eluted enantiomer.

**
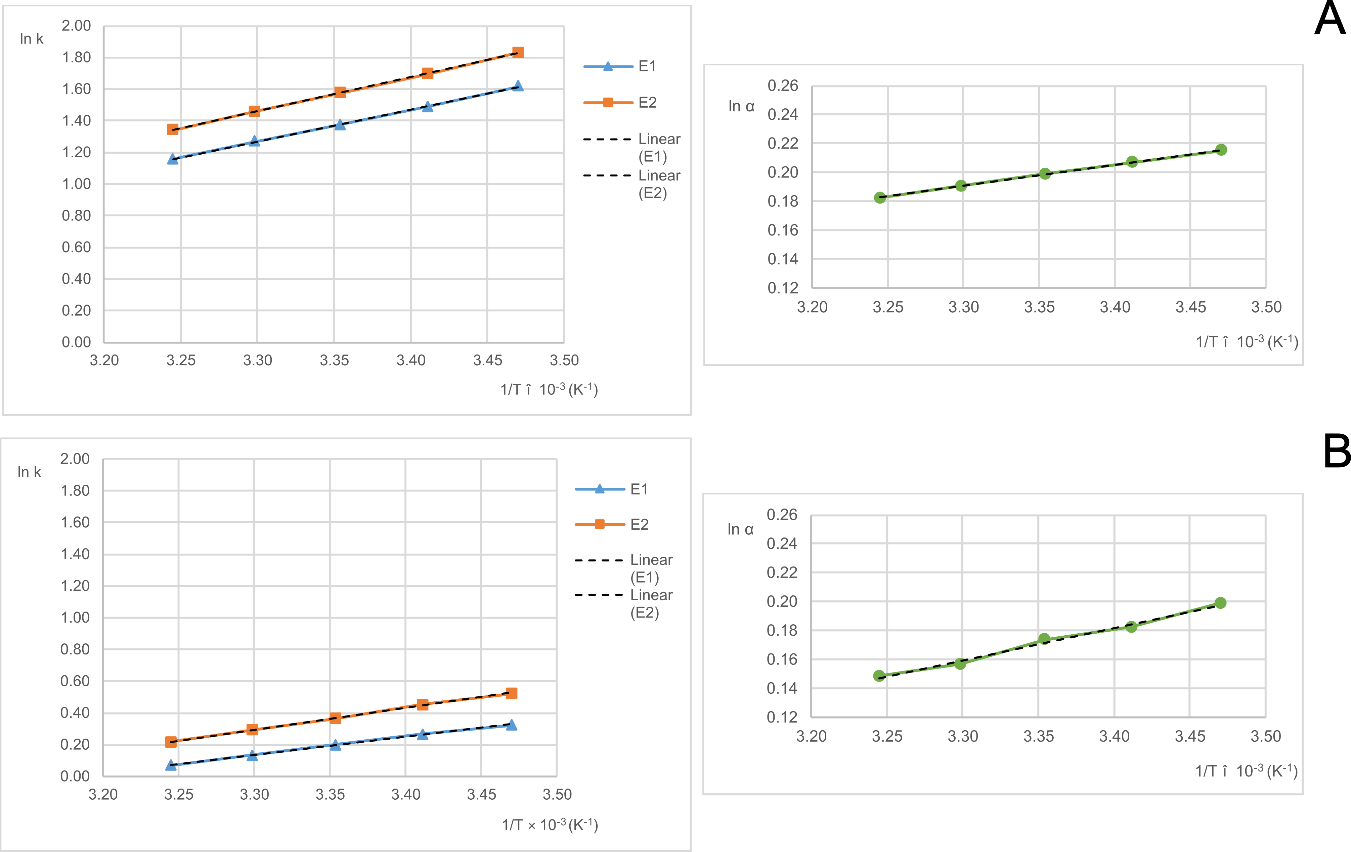
**

**Figure S1** - Van’t Hoff plots for the separation of NFX enantiomers in (A) Chiralpak^®^ AD CSP and (B) Chirobiotic V^®^ CSP. E1 = first-eluted enantiomer; E2 = second-eluted enantiomer.


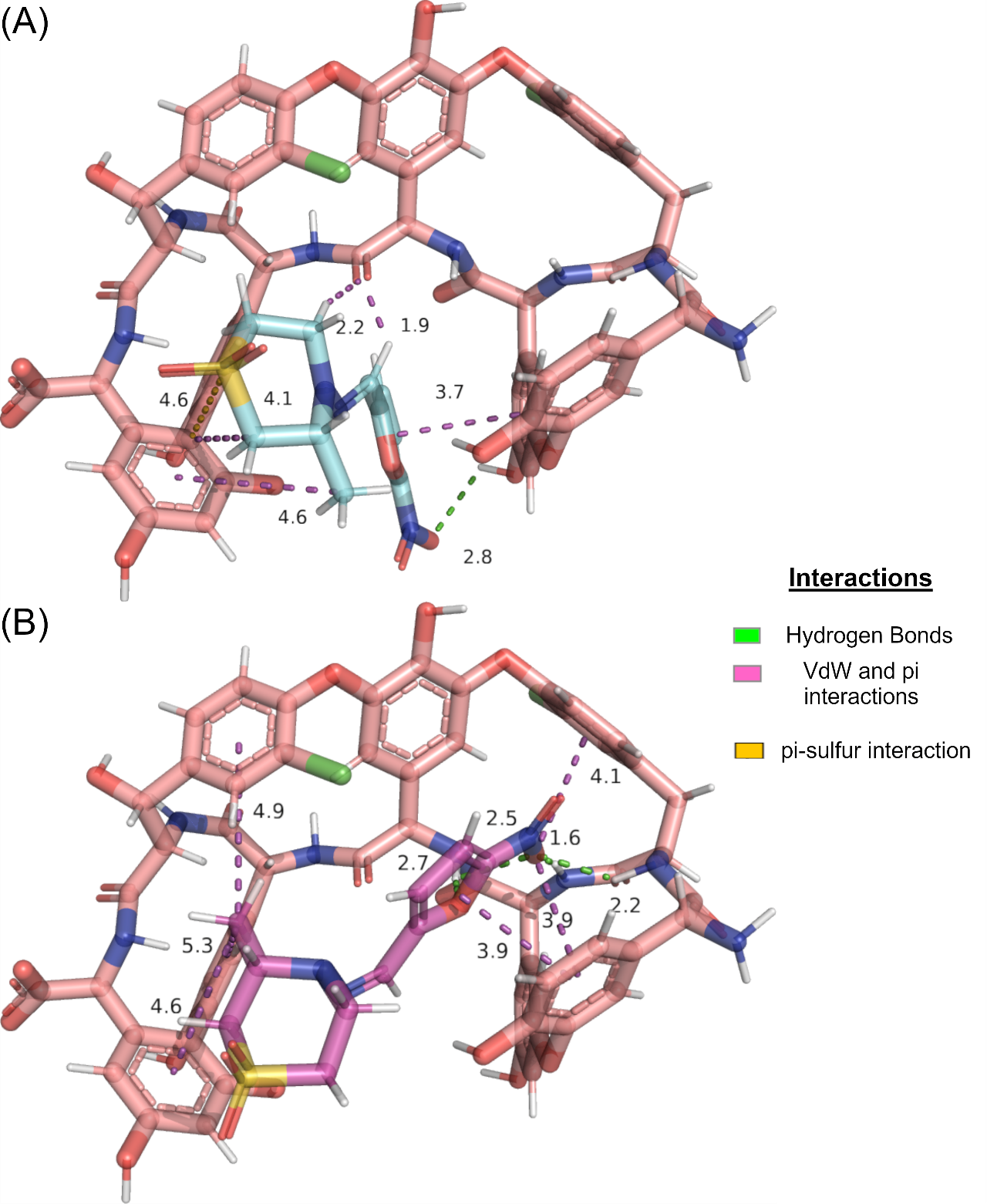


**Figure S2** - Interaction profile obtained for the best poses of NFX enantiomers in Chirobiotic^®^ T: (**A**) (R)-Nifurtimox; (**B**) (S)-Nifurtimox. Chirobiotic^®^ T CSP is represented with salmon carbon sticks, (R)-Nifurtimox enantiomer is represented in cyan carbon sticks and (S)-Nifurtimox enantiomer is represented in magenta carbon sticks. Hydrogen bond interactions are represented in green dashed lines, van der Waals and pi-interactions are represented in pink dashed lines and pi-sulfur interactions in yellow dashed lines.

**
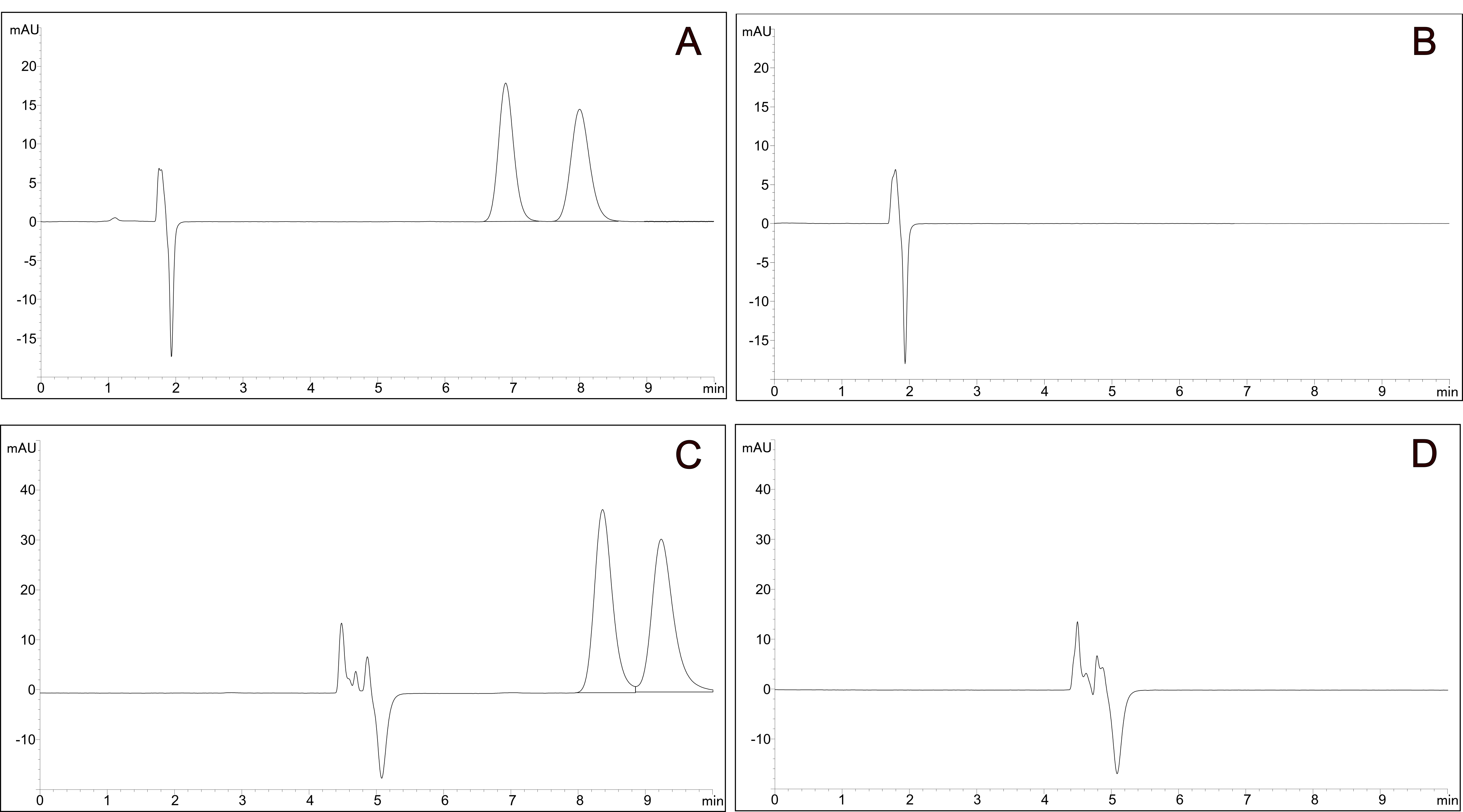
**

**Figure S3** - Illustrative chromatograms demonstrating the selectivity of the methodologies: (A) Chiralpak^®^ AD, NFX sample solution, (B) Chiralpak^®^ AD, placebo solution, (C) Chirobiotic V^®^, NFX sample solution (D) Chirobiotic V^®^, placebo solution.

**
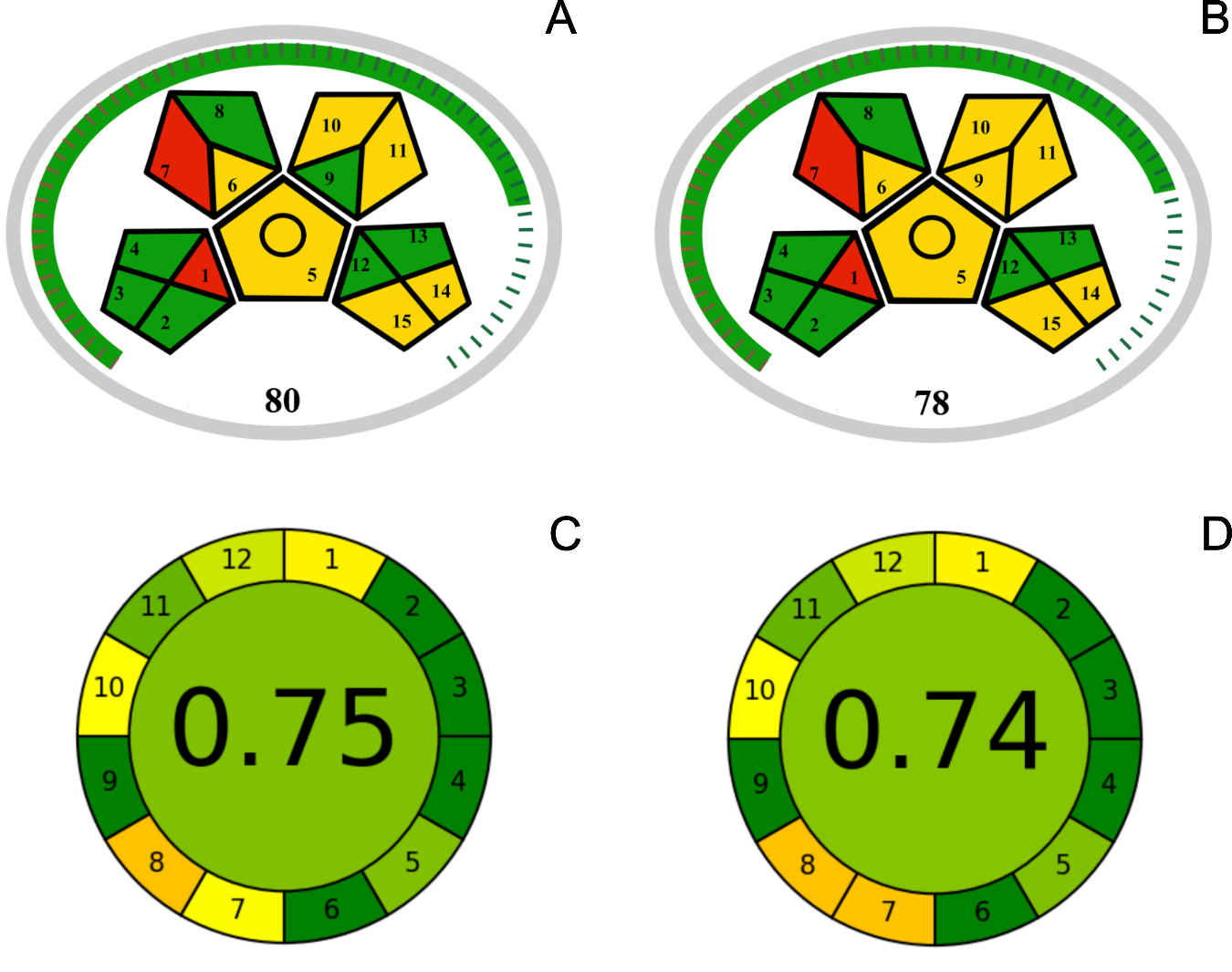
**

**Figure S4** - Environmental impact assessment of the developed methodologies: (A) MoGAPI pictogram of method in Chirobiotic^®^ V column, (B) MoGAPI pictogram of method in Chiralpak^®^ AD column, (C) AGREE pictogram of method in Chirobiotic^®^ V column, (D) AGREE method in Chiralpak^®^ AD column.
